# Dual Physically Crosslinked Hydrogels via Multi-Dimensional Carbon Materials for Methylene Blue Adsorption

**DOI:** 10.3390/gels12050452

**Published:** 2026-05-21

**Authors:** Yunxiang Zheng, Yonghan Wang, Mengmeng Wang, Xingzhou Wen, Chunxiao Zhang, Xiangpeng Wang

**Affiliations:** 1 School of Chemical Engineering, Shandong Institute of Petroleum and Chemical Technology, Dongying 257061, China; zhengyunxiang1990@163.com (Y.Z.); 15166282528@163.com (Y.W.); 2 Shandong Key Laboratory of Green Electricity & Hydrogen Science and Technology, Shandong Institute of Petroleum and Chemical Technology, Dongying 257061, China; zcx@sdipct.edu.cn; 3 PetroChina West–East Gas Pipeline Company, Shanghai 200120, China; wangmm03@pipechina.com.cn; 4 Lihuayi Group Co., Ltd., Dongying 257061, China; 15263851456@163.com; 5 College of Chemical Engineering and Environment, China University of Petroleum, Beijing 102249, China

**Keywords:** carbon nanomaterials, dual physically crosslinked, hydrogel, adsorption

## Abstract

The development of high-performance adsorbents for treating dye-laden wastewater necessitates a deep understanding of structure–property relationships. This study presents a systematic investigation into the role of carbon material dimensionality (0D biochar, BC; 1D carbon nanotubes, CNT; 2D graphene oxide, GO) in modulating the properties of a dual physically crosslinked sodium alginate/polyacrylamide (SA/PAM) hydrogel for methylene blue (MB) adsorption. A series of composite hydrogels was fabricated via a sequential physical crosslinking strategy. Comprehensive characterization confirmed the successful incorporation and dispersion of carbon materials within the dual network. The three hydrogels showed good mechanical properties. Under the conditions of 25 °C, an initial MB concentration of 100 mg/L, and pH 10–11, the incorporation of carbon materials enhanced the adsorption capacity, with maximum adsorption capacities of 411.5, 410.6, and 422.8 mg/g for BC-H, GO-H, and CNT-H, respectively. Coexisting constituents in real water samples reduce adsorption capacity via competitive adsorption and interfacial interference. After five consecutive adsorption–desorption cycles, the adsorption capacities of BC-H, GO-H, and CNT-H decreased to 57.7%, 67.2%, and 61.7% of their initial values, respectively. Adsorption isotherm and kinetic studies revealed that the process followed the Langmuir model and pseudo-second-order kinetics, indicative of monolayer chemisorption. Mechanistic analysis identified synergistic contributions from electrostatic attraction, π-π stacking, and physical entrapment. Physical structural changes and chemical site occupation are the main reasons for the decrease in the adsorption performance of hydrogels during cyclic use. This work provides a rational design strategy for advanced adsorbents and a theoretical foundation for efficient dye wastewater remediation.

## 1. Introduction

The treatment of industrial dye wastewater remains a significant environmental challenge. MB, a common cationic dye, is persistent in aquatic environments due to its stable aromatic structure and exhibits potential biotoxicity, posing substantial ecological risks [1]. Among various treatment technologies, adsorption is widely employed for its efficiency and cost effectiveness, spurring the development of novel adsorbents with high capacity and excellent recyclability [2]. In recent years, hydrogels have emerged as particularly promising adsorbents for dye removal, owing to their tunable three-dimensional polymeric networks, high water content, and modifiable physicochemical properties [3]. However, conventional single-network hydrogels are often limited by poor mechanical strength, swelling instability, and inadequate regeneration performance, which restricts their practical application [4].

To overcome these limitations, the construction of dual-network hydrogels through synergistic crosslinking strategies has proven effective [5]. Nevertheless, a single physically crosslinked network—whether based solely on ionic coordination or solely on hydrogen bonding—is often insufficient to simultaneously achieve robust mechanical strength, satisfactory swelling stability, and efficient regeneration capability [6]. Therefore, a dual functionalization approach that integrates both ionic and hydrogen bonding crosslinks within one hydrogel matrix becomes necessary [7]. Among these, systems based on SA and PAM are especially interesting. In these systems, multivalent cations (e.g., Fe^3+^, Ca^2+^) coordinate with SA’s guluronic units to form a stable “egg-box” ionic network, while hydrogen bonding between PAM and SA creates a flexible matrix. This combination of hydrogen and ionic bonding synergistically enhances both mechanical robustness and structural integrity, while the dynamic and reversible nature of these physical bonds facilitates efficient material regeneration [8,9].

Incorporating carbon-based nanomaterials offers a further route for enhancing hydrogel performance. GO, a 2D nanomaterial, provides abundant surface oxygen groups that act as multifunctional crosslinking sites and enable efficient dye adsorption via electrostatic interactions and π-π stacking [10]. CNTs, with their 1D structure and high aspect ratio, can form continuous transport pathways within the hydrogel, though their tendency to aggregate due to strong van der Waals forces remains a challenge that requires effective dispersion strategies [11]. BC, a cost-effective and eco-friendly 0D porous carbon derived from biomass, has gained attention for contributing abundant adsorption sites and serving as a sustainable filler [12]. While numerous studies have confirmed that incorporating single-type carbon materials can enhance hydrogel properties, recent research has increasingly focused on understanding how the intrinsic characteristics of these fillers, particularly their dimensionality, influence the final composite’s performance [13]. However, a systematic comparison of carbon materials with different dimensionalities within a single, well-defined dual physically crosslinking hydrogel matrix is still lacking. Such a comparative study is crucial for elucidating structure–property relationships and guiding the rational design of next-generation adsorbents.

Herein, we incorporate three carbon materials (GO, CNT, and BC) into an SA/PAM hydrogel system. By systematically varying the carbon type and content, this study investigates how carbon dimensionality influences their dispersion behavior and interfacial interactions within the hydrogen/ionic dual network, modulates the topological arrangement and dynamic properties of the hydrogel, and governs the evolution of the three-dimensional porous structure in relation to the MB adsorption mechanism. The work elucidates the structure–property relationships between multi-dimensional carbon materials and dual physically crosslinked hydrogels, offering not only a rational design strategy for high-performance adsorbents but also a theoretical and technical foundation for efficient dye wastewater treatment.

## 2. Results and Discussion

### 2.1. Preparation Mechanism Analysis

**Figure 1:**
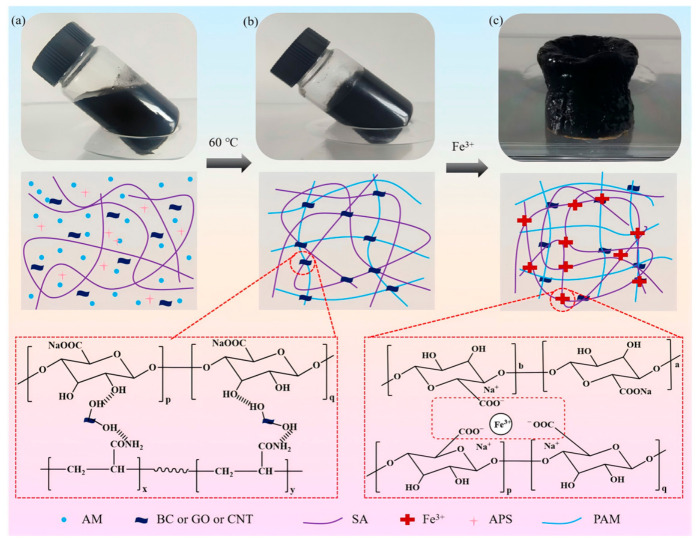
Preparation mechanism of dual physically crosslinked hydrogel. (**a**) Photos before aggregation, (**b**) after the first crosslinking, and (**c**) after the second crosslinking.

The dual physically crosslinked hydrogel was fabricated through a stepwise physical crosslinking strategy (Figure 1). The process begins with the formation of the first network: under thermal initiation, AM monomers undergo free-radical polymerization to form PAM chains. Simultaneously, carbon material act as multifunctional nano-crosslinkers, establishing extensive hydrogen bonds with both the PAM and SA chains via their surface oxygen-containing groups, resulting in an initial polymer–nanocomposite network. Subsequently, a second network is introduced for synergistic reinforcement: the primary hydrogel is immersed in an Fe^3+^ solution, where trivalent iron ions coordinate specifically with the guluronic units of SA chains, forming stable “egg-box” ionic crosslinks. Together, hydrogen bonding and ionic coordination constitute a dual physically crosslinked network. The hydrogen bonds contribute dynamic reversibility and energy dissipation, while the ionic crosslinks enhance mechanical strength and stability, collectively yielding a robust three-dimensional porous structure and superior macroscopic properties.

### 2.2. Characterization Analysis

**Figure 2:**
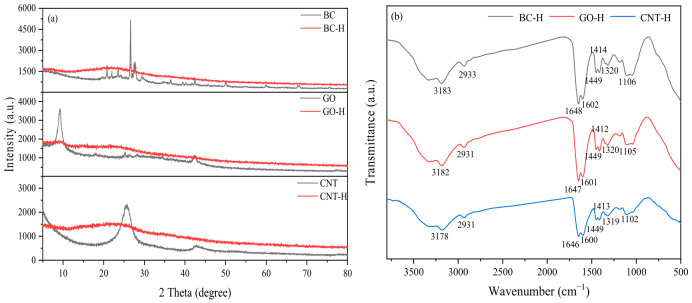
(**a**) XRD, (**b**) FT-IR spectra.

Figure 2a shows the XRD patterns of carbon materials and their composite hydrogels. In the pristine carbon materials, GO exhibits a typical (001) diffraction peak at around 10°, indicating an ordered layered structure [14]. CNT shows a characteristic (002) peak near 26°, corresponding to the graphitic stacking of carbon atoms [15]. In contrast, BC displays a broad diffuse peak at approximately 22–26°, consistent with its amorphous carbon nature [16]. Upon incorporation into the hydrogels, notable changes are observed in the XRD patterns: the GO diffraction peak weakens or disappears, suggesting effective exfoliation and dispersion within the hydrogel network. The CNT peak intensity decreases significantly, indicating polymer intercalation and successful debundling at the nanoscale. The broad peak of BC is largely retained, though minor shifts in intensity or position may occur, reflecting favorable physical integration with the polymer matrix. The attenuation or alteration of these characteristic peaks confirms the successful incorporation of carbon materials into the dual crosslinked network, rather than simple physical mixing [17]. The synthesis process effectively suppresses self-aggregation and promotes uniform dispersion and structural integration of the carbon materials within the hydrogel.

Figure 2b presents the FT-IR spectra of the three composite hydrogels. The highly consistent spectral features indicate that the incorporation of carbon materials does not alter the chemical bonding nature of the hydrogel system. Specifically, the broad peak at 3178–3183 cm^−1^ is attributed to O–H and N–H stretching vibrations. The absorption at 2931–2933 cm^−1^ corresponds to C–H stretching of –CH_2_– groups. The C=O stretching vibration of amide I appears at 1646–1648 cm^−1^, while the peaks at 1600–1602 cm^−1^ and 1449 cm^−1^ are assigned to asymmetric and symmetric stretching of –COO^−^, respectively. The signal at 1412–1414 cm^−1^ is associated with –COO^−^ vibrations in the guluronic units of alginate. Additionally, the absorption at 1319–1320 cm^−1^ belongs to amide III (C–N stretching coupled with N–H bending), and the broad peak around 1102–1106 cm^−1^ originates from C–O–C stretching in saccharide rings and C–OH stretching [18]. These spectral results collectively confirm the successful polymerization of polyacrylamide chains, the preservation of alginate carboxyl groups, and the dominance of physical interactions such as hydrogen bonding between carbon materials and the polymer matrix, with no significant covalent bonding formed. The absence of peak shifts with different carbon materials further suggests that carbon materials primarily act as nanoscale physical crosslinking points within the dual crosslinked network, without substantially modifying the chemical bonding environment.

**Figure 3:**
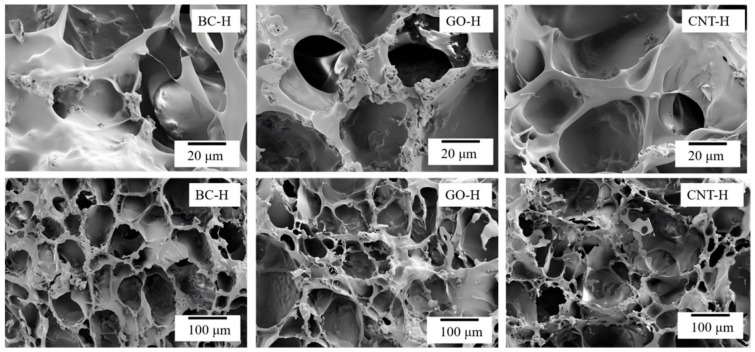
SEM images of composite hydrogels.

Figure 3 displays the SEM images of the three carbon-based composite hydrogels. All samples exhibit rough surfaces and a three-dimensional porous network structure. This characteristic morphology indicates the formation of well-developed internal pores within the hydrogels, providing effective transport pathways for dye molecule diffusion and abundant active sites for adsorption. Although different types of carbon materials were incorporated, no significant morphological differences are observed among the composite hydrogels. This suggests that under the given preparation conditions, the carbon materials primarily function as nano-reinforcing phases dispersed within the polymer matrix, without altering the fundamental formation mechanism or topology of the hydrogel network.

**Figure 4:**
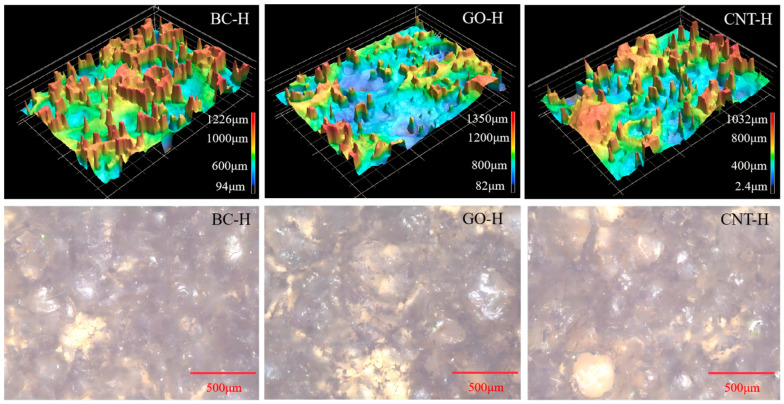
3D topology and 2D micromorphology of composite hydrogels.

Figure 4 presents the 3D morphology and corresponding 2D microstructures of the hydrogels. The composite hydrogels exhibit distinct hierarchical structural characteristics at the local scale. BC-H displays multi-scale protrusions with heights ranging from 94 to 1226 μm. Microscopically, BC particles are uniformly dispersed, collectively forming a rough surface and an interconnected porous network. GO-H shows more pronounced 3D undulations, with height variations spanning 82 to 1350 μm, featuring evident deep depressions and high protrusions. Its microstructure reveals localized aggregation of GO sheets, resulting in a porous yet heterogeneous phase distribution. In contrast, CNT-H exhibits relatively moderate height fluctuations (2.4–1032 μm), where CNTs are embedded as 1D fibrous structures within the polymer matrix. Although partial aggregation occurs, a continuous and relatively uniform three-dimensional network is formed. These observations demonstrate that carbon materials of different dimensions exhibit distinct assembly behaviors and spatial distribution characteristics within the dual crosslinked hydrogel network, providing crucial morphological insight for understanding their subsequent adsorption and mechanical properties.

### 2.3. Swelling Performance

**Figure 5:**
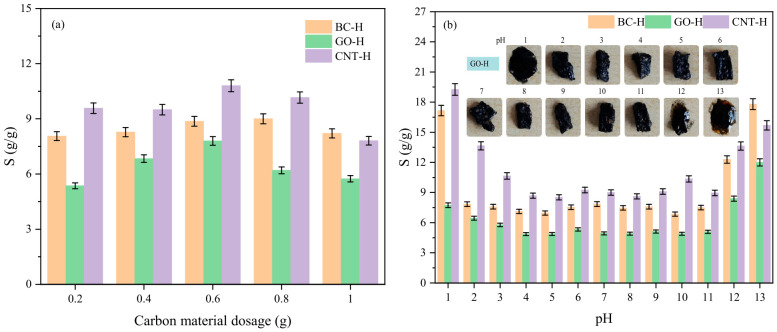
Effect of (**a**) carbon material dosage, (**b**) pH on the hydrogel swelling performance.

Figure 5a illustrates the effect of carbon material content on the swelling ratio. The swelling ratio initially increases and then decreases with increasing carbon content. At the same loading, the swelling capacity follows the order CNT-H > BC-H > GO-H. SEM results reveal similar microstructures among the three, indicating that the performance differences originate primarily from nanoscale interactions, with the key factor being the modulation of effective crosslinking density by the carbon materials [19]. At lower loadings, carbon materials act as hydrophilic fillers and physical crosslinkers, optimizing the network structure and enhancing hydrophilicity to promote swelling. In contrast, excessive carbon content leads to aggregation and serves as strong physical crosslinking points, significantly increasing crosslinking density and network rigidity, thereby restricting swelling. GO, with its abundant surface functional groups, forms dense hydrogen bonds with the polymer matrix, resulting in the strongest crosslinking effect and the lowest swelling ratio. CNTs, with their relatively inert surface, exhibit weak interactions and mainly provide structural support rather than strong crosslinking, leading to the highest swelling. BC exhibits intermediate surface properties and crosslinking efficiency, thus displaying moderate swelling behavior [20].

Figure 5b further reveals the influence of pH on the swelling behavior. The swelling ratio remains relatively low and stable within the pH range of 3–11, but increases significantly under strongly acidic or alkaline conditions. This behavior is governed by the combined effects of pH on the ionization state of functional groups (e.g., carboxyl and hydroxyl) and the stability of Fe^3+^ coordination bonds. Under extreme pH conditions, extensive protonation or deprotonation of surface groups introduces strong electrostatic repulsion, while the Fe^3+^ coordination structure becomes less stable. These factors synergistically relax and expand the hydrogel network, leading to a sharp increase in swelling. In contrast, across the neutral to weakly alkaline range, the hydrogel maintains a stable charge state and robust Fe^3+^ coordination, preserving a compact network structure and resulting in lower swelling ratios [21]. This pH-responsive behavior provides important theoretical guidance for the practical application of these hydrogels in real environments.

### 2.4. Mechanical Properties

**Figure 6:**
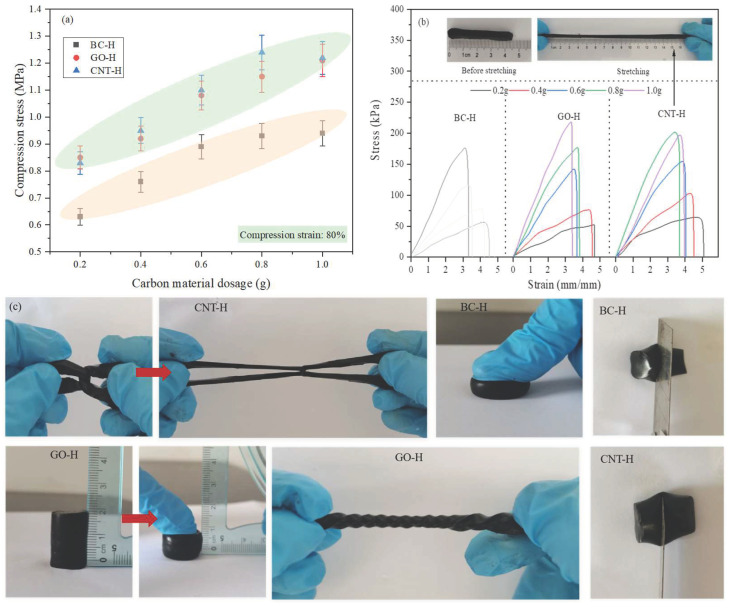
Mechanical characterization of hydrogels: (**a**) compression, (**b**) tension, and (**c**) corresponding physical demonstrations.

Figure 6 presents the mechanical test results of the composite hydrogels. Both tensile and compressive strengths increase significantly with higher carbon material loading, which can be attributed to the nanomaterials’ dual function as reinforcing fillers and physical crosslinking points. As their content increases, the dispersed carbon particles not only bear stress directly as rigid fillers but also effectively restrict polymer chain mobility through physical crosslinking. This synergistic effect substantially enhances the effective crosslinking density and network stability, resulting in improved modulus and strength at the macroscopic level.

The reinforcement efficiency, however, strongly depends on the carbon material type. At equivalent loadings, GO-H and CNT-H demonstrate markedly superior mechanical properties compared to BC-H, primarily due to fundamental differences in their nanostructures and interfacial characteristics. GO establishes extensive and robust interfacial bonding with polymer chains through its unique 2D layered structure and abundant oxygen-containing functional groups, enabling efficient stress transfer and distribution [22]. CNTs leverage their 1D fibrous morphology and high intrinsic modulus to function as “micro-rebar” reinforcements within the polymer matrix, providing bridging and pull-out effects that significantly dissipate fracture energy while simultaneously enhancing both strength and toughness [23]. In contrast, BC’s amorphous and isotropic porous structure, combined with its limited interfacial adhesion to the polymer matrix, results in less efficient stress transfer and consequently weaker reinforcement [24]. These findings collectively demonstrate that while the carbon content governs the magnitude of mechanical enhancement, the nanomaterials’ microstructure and interfacial properties ultimately determine both the mechanism and limits of performance optimization.

### 2.5. pH_pzc_ Analysis

**Figure 7:**
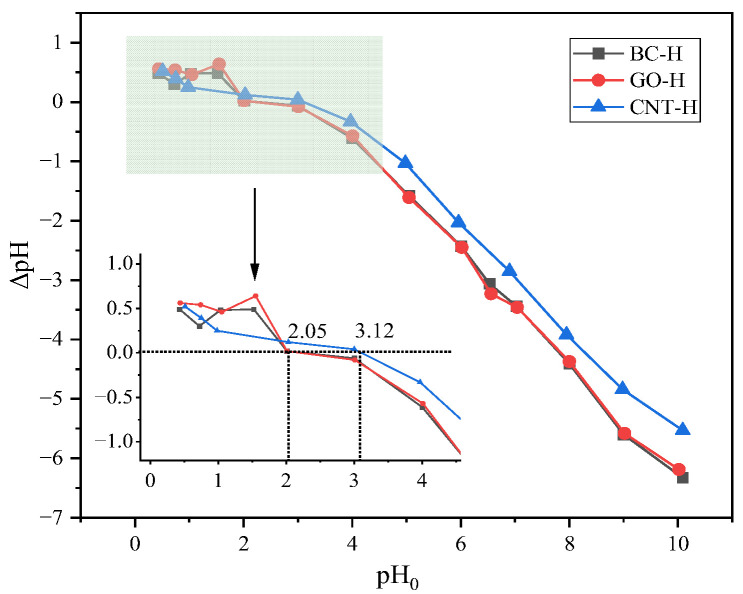
pH_pzc_ of composite hydrogels.

Figure 7 shows the pH_pzc_ of the three hydrogels determined by the pH drift method. Both BC-H and GO-H exhibit a pH_pzc_ of 2.05, while CNT-H shows a relatively higher value of 3.12. The pH_pzc_ represents the critical point where the net surface charge of the material is zero. At solution pH below the pH_pzc_, the hydrogel surface becomes protonated and carries a positive charge; above this value, deprotonation occurs, resulting in a negative surface charge. Thus, across a wide pH range well above their respective pH_pzc_ values, all three hydrogels possess a negatively charged surface.

The differences in pH_pzc_ among the hydrogels are attributed to their distinct chemical compositions. BC-H and GO-H likely contain more abundant acidic oxygen-containing functional groups, which deprotonate at relatively low pH, leading to their lower pH_pzc_. In contrast, CNT-H possesses fewer such acidic groups or more basic sites, requiring a higher pH to achieve a net negative surface charge. Given that MB is a cationic dye, its adsorption is expected to be strongly driven by electrostatic attraction. These surface charge characteristics provide essential electrochemical insight for interpreting the variations in adsorption capacity and behavior among the different hydrogels.

### 2.6. Adsorption Performance

**Figure 8:**
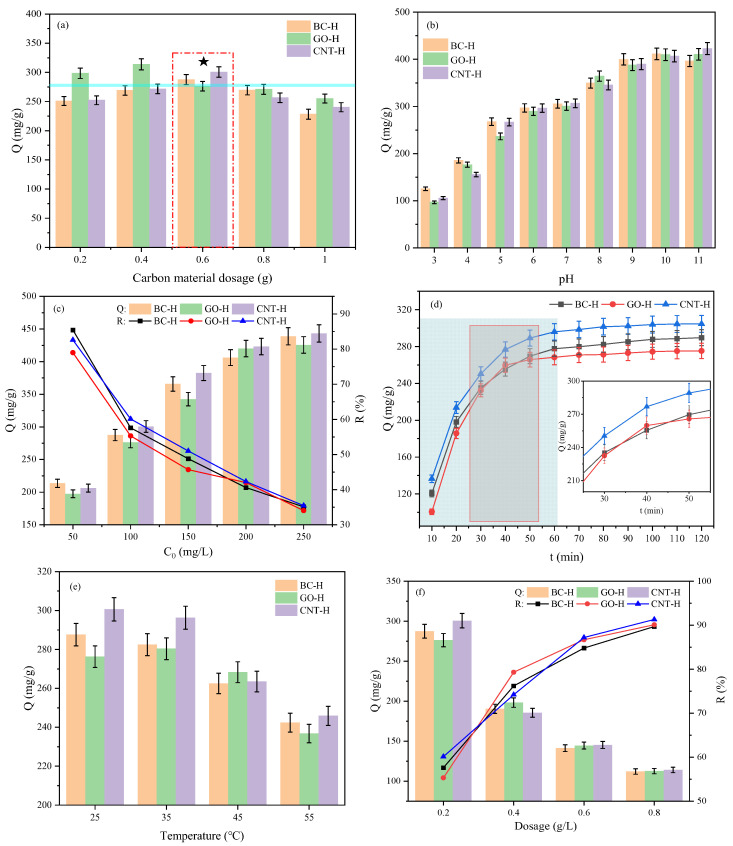
Effect of (**a**) carbon material content, (**b**) pH, (**c**) initial MB concentration, (**d**) contact time, (**e**) temperature, and (**f**) dosage on hydrogel adsorption.

To quantify the synergistic effect, controlled experiments with pure GO were conducted under identical conditions. Pure GO exhibits an adsorption capacity of 186.5 mg/g for MB, whereas all GO-H hydrogels display significantly higher adsorption capacities (Figure 8a). This enhancement confirms that the three-dimensional physical crosslinking network effectively prevents GO restacking and provides additional adsorption sites, thereby synergistically improving adsorption performance.

Figure 8a reveals the effect of carbon content on the MB adsorption capacity. The adsorption capacities of BC-H, GO-H, and CNT-H all initially increase and then decrease with increasing carbon loading. Specifically, CNT-H and BC-H reach their maximum adsorption capacities at 0.6 g, with values of 287.6 mg/g and 300.6 mg/g, respectively. In contrast, GO-H achieves its optimal capacity of 313.8 mg/g at a lower loading of 0.4 g. This trend is attributed to the dual role of carbon materials: at moderate loadings, they provide more active sites for adsorption, but excessive amounts lead to nanoparticle aggregation, which blocks accessible sites and increases mass transfer resistance, ultimately reducing performance [25]. Notably, the lower optimal loading for GO-H can be ascribed to the 2 D structure and abundant oxygen-containing groups of GO, which facilitate better dispersion and expose active sites more efficiently at lower contents; beyond 0.4 g, the restacking of GO sheets likely occurs, causing a sharper decline in capacity compared to the 1 D CNT and 0 D BC. Based on these results, 0.6 g was selected as the optimal loading for subsequent experiments, balancing performance and cost-effectiveness.

Figure 8b illustrates the influence of solution pH on adsorption behavior. The MB adsorption capacity of all three hydrogels increases significantly as the pH shifts from acidic to alkaline, reaching a maximum in the pH 10–11 range. This pattern strongly correlates with the surface charge properties of the hydrogels. As previously determined, BC-H and GO-H have a pH_pzc_ of 2.05, while CNT-H exhibits a pH_pzc_ of 3.12. Thus, across most of the tested pH range, particularly under neutral to alkaline conditions, the hydrogel surfaces are negatively charged, facilitating efficient electrostatic attraction of cationic MB molecules. In acidic conditions (pH < pH_pzc_), the surfaces become protonated and positively charged, leading to electrostatic repulsion and competition with H^+^ ions, which explains the low adsorption. The relatively lower adsorption capacity of GO-H within the pH 3–7 range, compared to the other composites, can be attributed to the more pronounced protonation of its surface carboxylic groups, which are present in higher density on GO. This further confirms the role of specific functional groups in pH-dependent adsorption [26]. Higher pH values promote greater deprotonation of acidic functional groups, enhancing surface negativity and thereby increasing adsorption capacity.

Figure 8c examines the effect of initial MB concentration. The equilibrium adsorption capacity increases monotonically with higher initial concentrations, while the removal efficiency gradually declines. This behavior follows fundamental adsorption principles: higher concentrations provide a greater driving force (concentration gradient), promoting diffusion and occupancy of available sites until saturation is reached. However, since the number of active sites is limited for a fixed adsorbent dosage, the removal efficiency inevitably decreases as the initial concentration rises [27]. The plateau observed at high concentrations indicates progressive saturation of binding sites; the differences in the maximum uptake among the three composites likely arise from variations in accessible surface area and functional group density imparted by the dimensionality of the carbon fillers.

Figure 8d presents the adsorption kinetics. All three hydrogels exhibit a similar adsorption process: an initial rapid phase (within 60 min), followed by a slower approach to equilibrium. The rapid stage is driven by the abundance of surface sites and large concentration gradient, enabling fast diffusion and attachment of dye molecules to the surface and macropores. As surface sites become occupied, dye molecules must diffuse further into the gel network, increasing mass transfer resistance and slowing the adsorption rate until equilibrium is established.

As shown in Figure 8e, temperature has a significant effect on the adsorption capacity of the three hydrogels for MB. In the temperature range of 25–35 °C, all three hydrogels maintain relatively high adsorption capacities; as the temperature further increases to 45–55 °C, the adsorption capacities gradually decrease. This trend indicates that increasing temperature is unfavorable for the adsorption process, suggesting that the adsorption of MB by the three hydrogels is likely an exothermic reaction.

As displayed in Figure 8f, with an increase in hydrogel dosage, the adsorption capacity of the three hydrogels for MB gradually decreases, while the removal rate shows an increasing trend. At higher dosages, although the total number of adsorption sites increases, the amount of dye adsorbed per unit mass of hydrogel decreases due to the “site dilution effect”, and some active sites are not fully saturated. Meanwhile, the removal rate increases with higher dosage because more adsorbent provides a greater abundance of surface functional groups and binding sites, resulting in a significant reduction in the residual concentration of methylene blue in solution.

**Figure 9:**
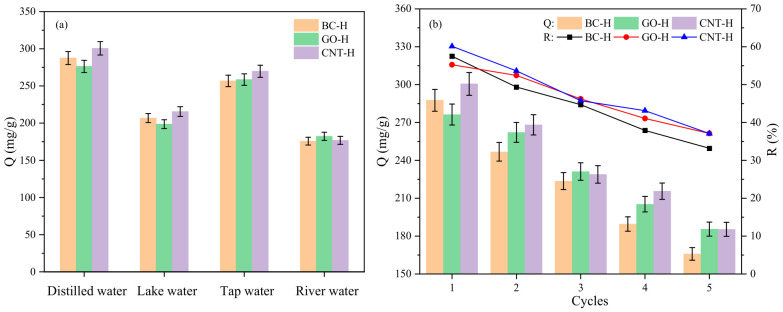
(**a**) Adsorption performance in simulated wastewater, (**b**) reusability.

Figure 9a evaluates the adsorption performance of the three hydrogels toward MB in different water matrices, including distilled water, lake water, tap water, and river water. The results indicate that all hydrogels achieve the highest adsorption capacity in distilled water, while exhibiting varying degrees of reduction in real water samples. This decline is primarily attributed to the competitive adsorption and interfacial interference caused by common coexisting constituents in natural water environments—such as inorganic salts, dissolved organic matter, and suspended particles. These components not only compete for active sites on the hydrogel surface but may also alter the swelling behavior and surface charge distribution of the hydrogel network, thereby affecting the mass transfer efficiency of dye molecules and the final adsorption equilibrium.

Figure 9b further examines the regeneration performance and stability of the hydrogels over five consecutive adsorption–desorption cycles. The adsorption capacity and removal efficiency of all samples gradually decline with increasing cycles. Specifically, after five cycles, the adsorption capacities of BC-H, GO-H, and CNT-H decreased from their initial values of 287.6, 276.3, and 300.6 mg/g to 165.9, 185.6, and 185.4 mg/g, respectively. This performance loss can be ascribed to the irreversible occupation or collapse of some active sites during cycling, as well as physical damage to the hierarchical pore structure caused by repeated regeneration, collectively leading to a progressive reduction in effective surface area and adsorption activity.

In summary, although the adsorption capacities of BC-H, GO-H, and CNT-H for MB vary under different environmental conditions, their overall performance remains within the same order of magnitude, confirming the adsorption potential of all three composites. Specifically, GO-H likely benefits from the abundant oxygen-containing functional groups on GO, which provide ample electrostatic adsorption sites and enhanced hydrophilicity. CNT-H presumably leverages the three-dimensional network and hollow tubular structure formed by intertwined CNTs, endowing the hydrogel with well-developed mesoporous channels that facilitate MB mass transfer and capillary condensation. Meanwhile, BC-H combines the inherent porous structure of BC with the structural resilience of the gel network, achieving an effective balance between specific surface area and mechanical stability. Thus, although the three carbon materials modulate and optimize the physical structure and chemical environment of the hydrogels in distinct ways, they all contribute synergistically to the capture and retention of MB molecules, resulting in comparably high adsorption performance with only minor variations. This finding highlights that the final properties of hydrogels can be effectively tailored through appropriate carbon material selection to meet the requirements of specific adsorption applications.

### 2.7. Adsorption Theoretical Analysis

#### 2.7.1. Adsorption Isotherm

To further understand the adsorption behavior of the composite hydrogels toward MB, the experimental data were fitted using the Langmuir (Equation (1)) and Freundlich (Equation (2)) models [28].

(1) $$\frac{C_{e}}{Q_{e}}=\frac{1}{Q_{m}K_{b}}+\frac{C_{e}}{Q_{m}}$$

(2) $${\mathrm{lnQ}}_{e}={\mathrm{lnK}}_{f}+\frac{1}{n}{\mathrm{lnC}}_{e}$$

(3) $$R_{L}=\frac{1}{1+{\mathrm{KbC}}_{0}}$$

where C_e_ denotes the equilibrium concentration of MB (mg/L), Q_e_ represents the equilibrium adsorption capacity (mg/g), Q_m_ is the maximum theoretical adsorption capacity (mg/g), K_b_ is the Langmuir adsorption constant (L/mg), K_f_ is the Freundlich adsorption constant (g^−1^·mg^1−1/n^·L^−1/n^), and n is the adsorption intensity parameter. The dimensionless separation factor R_L_, a parameter derived from the Langmuir model, is defined by Equation (3), where C_0_ (mg/L) is the initial MB concentration. An R_L_ > 1 suggests unfavorable adsorption, R_L_ = 1 indicates linear adsorption, 0 < R_L_ < 1 signifies favorable adsorption, and R_L_ = 0 implies irreversible adsorption.

**Figure 10:**
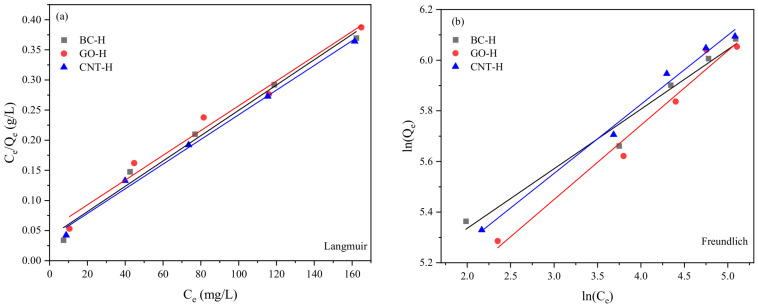
Fitting curves of (**a**) Langmuir and (**b**) Freundlich models.

As shown in Figure 10 and Table 1, the correlation coefficients (R^2^) for the Langmuir model are higher than those for the Freundlich model across all three hydrogels, indicating that the adsorption of MB follows the Langmuir model more closely and is characteristic of monolayer adsorption. This implies that the adsorption is dominated by a homogeneous distribution of binding sites with uniform adsorption energy, and that intermolecular interactions between the adsorbed MB molecules are negligible under the tested conditions. Furthermore, the calculated R_L_ values all fall within the range of 0 to 1, confirming that the adsorption process is favorable. A closer examination of the R_L_ values reveals that they decrease with increasing initial MB concentration—a trend consistent with the Langmuir isotherm behavior, meaning that the adsorption becomes more thermodynamically favorable at higher concentrations due to the stronger driving force for site occupation. The 1/n values obtained from the Freundlich fitting are all less than 1, which also supports the favorability of adsorption. However, the consistently lower R^2^ values for the Freundlich model compared to the Langmuir model suggest that the contribution of heterogeneous multilayer adsorption is limited, and that the monolayer adsorption mechanism described by the Langmuir model plays a predominant role in this system.

#### 2.7.2. Adsorption Kinetics

The adsorption kinetic data were fitted using the pseudo-first-order (Equation (4)), pseudo-second-order (Equation (5)), intra-particle diffusion (Equation (6)) and the liquid film (Equation (7)) diffusion models [28].

(4) $$\log(Q_{e}-Q_{t})=-\frac{k_{1}}{2.303}t+\log Q_{e}$$

(5) $$\frac{t}{Q_{t}}=\frac{t}{Q_{e}}+\frac{1}{k_{2}}\frac{1}{Q_{e}^{2}}$$

(6) $$Q_{t}{=\mathrm{Kt}}^{\frac{1}{2}}+C$$

(7) $$\ln(1-\frac{Q_{t}}{Q_{e}})={\;C}_{1}-k_{\mathrm{FD}}t$$

where Q_t_ represents the adsorption capacity of the hydrogel for MB at a specific time t, mg/g; t denotes the duration of the adsorption process in minutes; Q_e_ stands for the equilibrium adsorption capacity, mg/g; and k_1_ and k_2_ are the pseudo-first-order and pseudo-second-order adsorption rate constants, respectively; K is the intra-particle diffusion rate constant and C reflects the boundary layer effect; and k_FD_ is diffusion rate constant (1/min) and C_1_ is constant.

**Figure 11:**
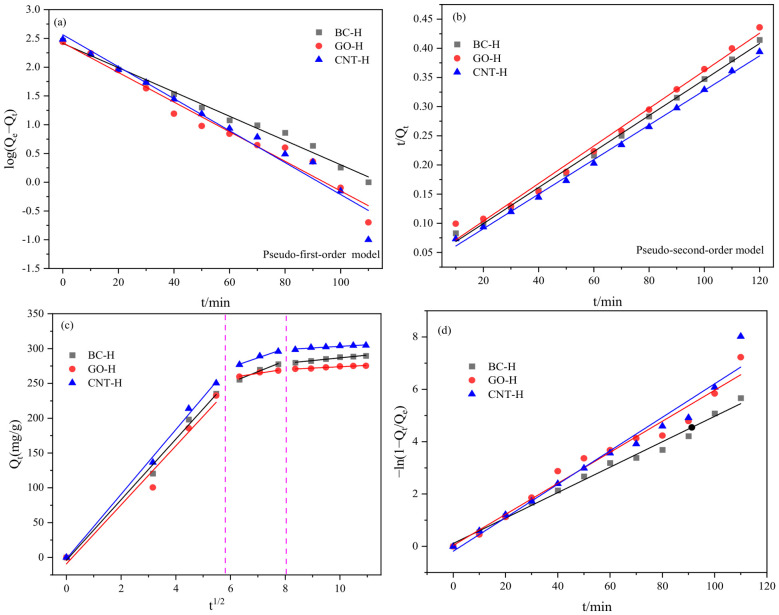
Adsorption kinetics curves of hydrogels on MB: (**a**) pseudo-first-order, (**b**) pseudo-second-order, (**c**) intra-particle diffusion model, (**d**) liquid film diffusion model.

Figure 11 shows the fitting curves of the pseudo-first-order and pseudo-second-order models for MB adsorption, with corresponding parameters listed in Table 2. The higher correlation coefficients (R^2^) of the pseudo-second-order model, along with its calculated equilibrium capacities (BC-H: 323.62 mg/g, GO-H: 310.56 mg/g, CNT-H: 337.84 mg/g) being closer to the experimental values (BC-H: 289.6 mg/g, GO-H: 275.3 mg/g, CNT-H: 304.6 mg/g), indicate that this model better describes the adsorption process, suggesting chemisorption as the dominant mechanism.

The intra-particle diffusion model was applied to elucidate the rate-controlling steps, and the plots exhibit multi-linearity, indicating a three-stage adsorption process with decreasing rate constants (K_1_ > K_2_ > K_3_). The first stage represents film diffusion, where MB molecules rapidly migrate to the external surface of the composite hydrogels. The second stage corresponds to intra-particle diffusion into the interior of the dual-network structure, where MB molecules gradually penetrate through macropores and interact with the polymer mesh. The third stage is the final equilibrium stage, where diffusion slows as active sites become saturated and MB concentration decreases [29]. This three-stage progression confirms that adsorption on the composite hydrogels follows a fast initial surface uptake, followed by slower intra-particle diffusion until equilibrium is reached. Meanwhile, the fitting line of the liquid film diffusion model also does not pass through the origin of the coordinates. Therefore, the diffusion of MB dye molecules is not only controlled by liquid film diffusion alone, but by the combination of liquid film diffusion and intra-particle diffusion.

#### 2.7.3. Adsorption Thermodynamics

**Figure 12:**
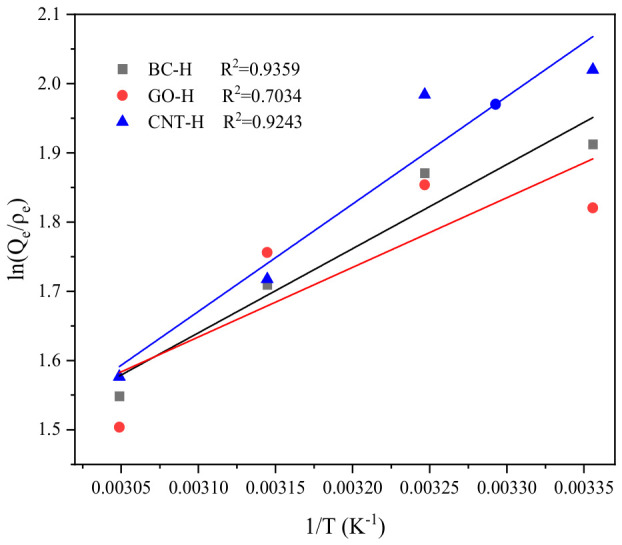
Van’t Hoff curves.

To study the adsorption thermodynamics, the free energy change (ΔG), enthalpy change (ΔH), and entropy change (ΔS) are calculated, and the calculation formulas are shown in Equations (8) and (9), respectively. The van’t Hoff curves and thermodynamic parameters are displayed in Figure 12 and Table 3, respectively.

(8) $$\ln\frac{Q_{e}}{\rho_{e}}=\frac{\Delta S}{R}-\frac{\Delta H}{\mathrm{RT}}$$

(9) ΔG=ΔH−TΔS

where ρ_e_ is the MB concentration after adsorption, mg/L; Q_e_ is the equilibrium adsorption capacity, mg/g.

The data in the table indicate that negative ΔG values imply that the adsorption of MB by the three hydrogels is spontaneous in all cases. Furthermore, the ΔG increases with rising temperature, indicating that the adsorption system exhibits stronger spontaneity at lower temperatures. The negative ΔH suggests that the adsorption process of this system releases heat. The negative ΔS indicates a decrease in the free energy at the solid–liquid interface during the adsorption process.

#### 2.7.4. Analysis of Adsorption Mechanism and Cyclic Efficiency Decay

The adsorption of MB onto BC-H, CNT-H, and GO-H all conformed excellently to the Langmuir isotherm model and the pseudo-second-order kinetic model. This uniformity implies that the underlying adsorption mechanisms, namely monolayer chemisorption, are fundamentally similar for all three hydrogels. Consequently, a detailed investigation of GO-H is expected to provide representative insights into the chemical interactions governing the adsorption process for this class of materials.

**Figure 13:**
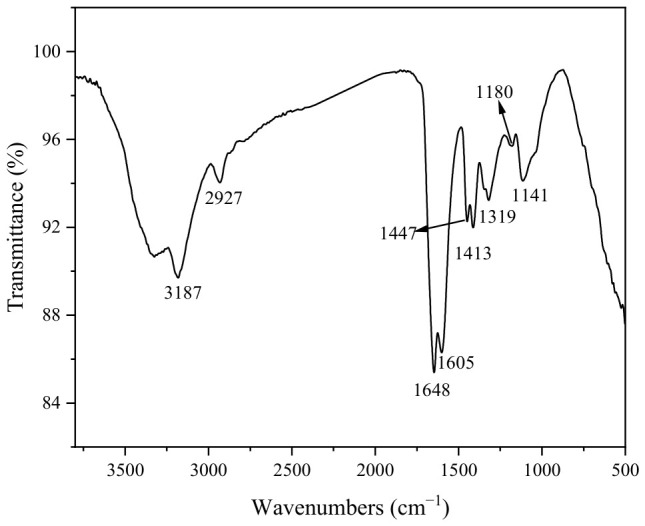
FTIR spectrum of GO-H after MB adsorption.

Figure 13 displays the FTIR spectrum of GO-H after MB adsorption. Compared to the spectrum of pristine GO-H (Figure 2b), the broad peak at 3182 cm^−1^, attributed to O-H or N-H stretching vibrations, shifts to 3187 cm^−1^ after adsorption, suggesting enhanced hydrogen bonding or structural rearrangement. The C-H stretching vibration at 2931 cm^−1^ redshifts to 2927 cm^−1^, likely due to hydrophobic interactions or changes in the local polar environment. The peaks at 1647 cm^−1^ and 1601 cm^−1^, corresponding to the amide I band (C=O) and aromatic rings or carboxylate groups, respectively, show little change for the former (1648 cm^−1^) while the latter shifts to 1605 cm^−1^. This implies that the aromatic rings or carboxyl groups participate in π-π stacking or electrostatic interactions with the aromatic structure of MB. The peaks at 1449 cm^−1^ and 1412 cm^−1^ shift to 1447 cm^−1^ and 1413 cm^−1^, respectively, further confirming the involvement of carboxyl groups in the adsorption process. Notably, the band at 1105 cm^−1^ (C-O-C or C-OH) disappears, while two new peaks emerge at 1180 cm^−1^ and 1141 cm^−1^. This is likely due to the formation of new hydrogen bonds or coordination bonds between the nitrogen-containing groups of MB and the oxygen-containing functional groups of the hydrogel, altering the vibrational modes of the original C-O bonds [30]. In summary, the observed shifts in the FTIR spectra indicate that the adsorption mechanism involves hydrogen bonding, π-π stacking, and electrostatic interactions.

**Figure 14:**
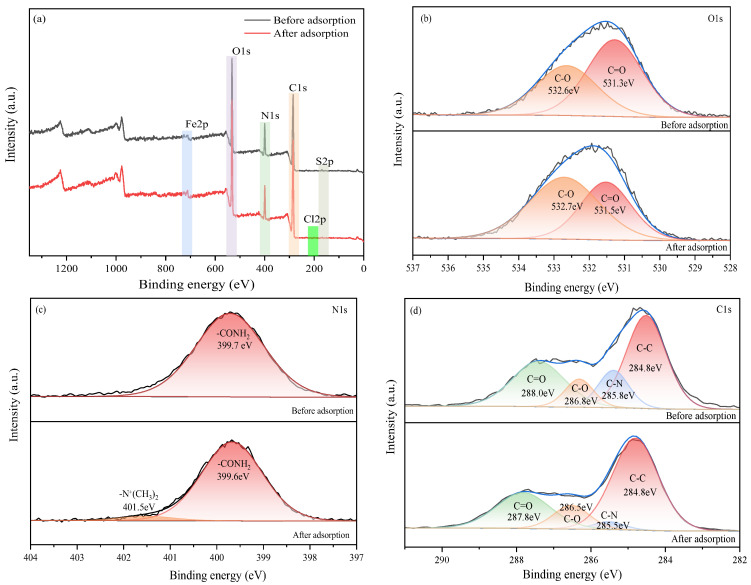
XPS spectrums: (**a**) survey scan, (**b**) O1s, (**c**) N1s, (**d**) C1s of hydrogels before and after MB adsorption.

Comparative XPS analysis before and after MB adsorption provides key insights into the interaction mechanisms between surface functional groups and dye molecules. The survey scan (Figure 14a) reveals the emergence of a Cl 2p peak at approximately 200 eV after adsorption, confirming successful MB loading on the hydrogel surface. Detailed examination of the O 1s spectrum (Figure 14b) shows that the pre-adsorption profile could be deconvoluted into oxygen-containing functional groups at approximately 532.6 eV (C–O) and 531.3 eV (C=O). Post-adsorption, these peaks exhibit binding energy shifts and significant intensity reduction, indicating that hydroxyl and carboxyl groups participate in the adsorption process through electrostatic attraction or coordination with MB molecules. The N 1s spectrum (Figure 14c) demonstrates a notable transformation: while only the amide nitrogen (–CONH_2_) from polyacrylamide is observed at approximately 399.7 eV before adsorption, a new peak appears at approximately 401.5 eV after MB uptake, characteristic of the positively charged quaternary ammonium group (–N^+^(CH_3_)_2_) in MB molecules. This provides direct evidence for electrostatic interaction between cationic MB and negatively charged surface groups on GO-H. Furthermore, the C 1s spectrum (Figure 14d) indicates decreased signals from C–O and O–C=O functional groups after adsorption, while the enhanced aromatic carbon signature suggests π-π stacking interactions between MB molecules and GO sheets [31].

**Figure 15:**
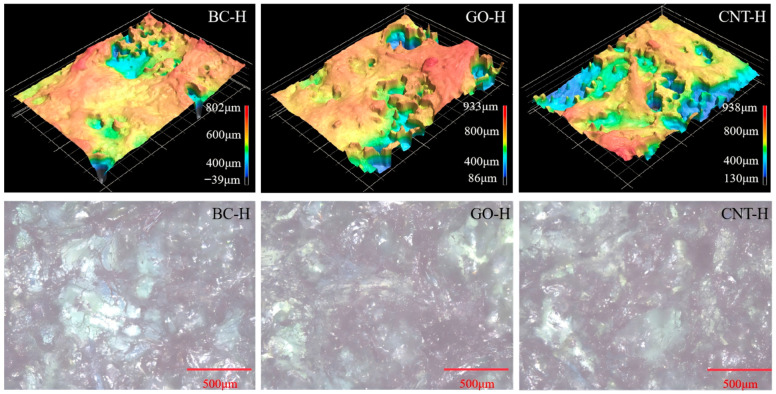
3D topology and 2D micromorphology of composite hydrogels after MB adsorption.

Figure 15 shows CLSM images of the hydrogels after MB adsorption. The deep blue color visually confirms MB accumulation both on the surface and within the hydrogel matrix. The 3D topography exhibits reduced elevation, decreased surface roughness, and diminished pore structure, which collectively reflect the infiltration and filling of MB molecules within the hydrogel network. Through synergistic mechanisms including physical entrapment, electrostatic attraction, and π-π stacking, MB molecules penetrate the pore structure and adsorb onto the hydrogel surface and network junctions. This process leads to pore filling, surface smoothing due to MB coverage, and a consequent decrease in elevation, while the concentrated MB gives the surface a deep blue appearance. The multidimensional changes, characterized by pore filling, surface coverage, and color development, provide structural and visual evidence supporting the cooperative adsorption mechanism between MB and the hydrogel. The porous or interwoven network constructed by the carbon materials facilitates mass transfer and provides adsorption sites. The adsorption behavior not only alters the three-dimensional topology of the hydrogel but also visualizes the adsorption process, offering direct support from a visualization perspective for the proposed mechanisms of electrostatic attraction, π-π stacking, and physical filling.

The BET and BJH parameters of GO-H before and after MB adsorption are presented in Table 4. Following MB adsorption, the specific surface area, total pore volume, BJH cumulative pore volume, and average pore diameter of the GO-H hydrogel all exhibit a significant decreasing trend, indicating that dye molecules successfully occupy the active sites on the hydrogel surface and fill the pore channels, leading to partial pore blockage. After four adsorption–desorption cycles, these structural parameters further decrease markedly, which is mainly attributed to sustained pore blockage caused by irreversible adsorption and partial collapse of the hydrogel skeleton induced by repeated swelling–drying processes. The continuous reduction in pore diameter particularly reflects the “small-pore blocking” mechanism, where smaller mesopores or pore throats are preferentially occupied.

**Figure 16:**
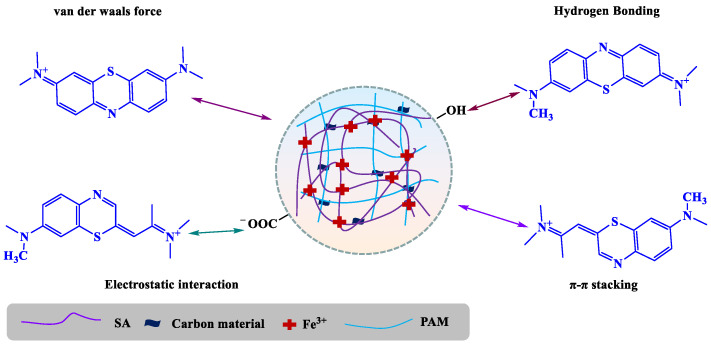
Proposed mechanism for MB adsorption.

In summary, as shown in Figure 16, the adsorption mechanism involves multiple synergistic contributions: electrostatic attraction between oxygen-containing functional groups (particularly carboxyl) on GO-H and MB cations, π-π stacking between aromatic structures, structural support from the Fe^3+^ crosslinked coordination network, and additional van der Waals interactions. These combined mechanisms enable highly efficient MB adsorption by the GO-H composite material.

**Figure 17:**
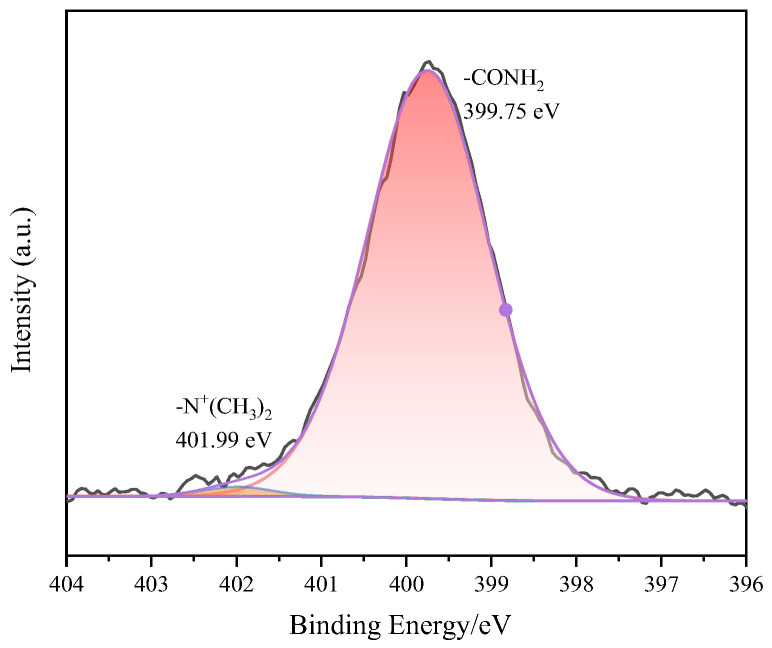
N1s spectrum of GO-H after four adsorption–desorption cycles.

Additionally, the reasons for the decrease in adsorption performance of GO-H after multiple reuse cycles were thoroughly analyzed. As presented in Table 4, both the specific surface area and pore size of GO-H decreased to a certain extent after four adsorption–desorption cycles. This change suggests that the repeated adsorption process may lead to partial pore blockage by residual dye molecules or local collapse of the three-dimensional network structure of the hydrogel, thereby restricting the diffusion of adsorbates to internal active sites. Concurrently, XPS analysis in Figure 17 detected a characteristic peak at 401.99 eV in the N1s spectrum of the cycled sample, corresponding to the quaternary ammonium nitrogen of methylene blue. The appearance of this peak provides direct evidence that, despite the desorption treatment, a small amount of dye molecules or their fragments remained within the gel matrix, occupying active sites originally available for adsorption. Based on these results, the decline in the cyclic usage efficiency of GO-H can be attributed to the combined effects of physical structural changes and chemical site occupation.

### 2.8. Comparison with Other Adsorbents

To evaluate the practical potential of the composite hydrogels for MB removal, Table 5 presents a comparative analysis of the adsorption capacities of various carbon-based hydrogel adsorbents. The maximum adsorption capacities of BC-H, GO-H, and CNT-H for MB reach 411.5, 422.8, and 410.6 mg/g, respectively. Compared with other carbon-based hydrogel adsorbents reported in the literature, the performance of our materials is competitive. The inclusion of detailed experimental conditions in Table 4 allows for a more meaningful comparison, confirming that the developed hydrogels have promising potential for practical dye removal applications.

## 3. Conclusions

This study systematically investigated the role of carbon material dimensionality (0D BC, 1D CNT, and 2D GO) in modulating the properties of dual physically crosslinked SA/PAM hydrogels for MB adsorption. The findings demonstrate that carbon dimensionality is a critical design parameter that governs the nanoscale interactions, dispersion behavior, and assembly of additives within the dual-network matrix, ultimately dictating the hydrogel’s crosslinking density, pore structure evolution, and interfacial affinity. The incorporation of carbon materials significantly enhanced adsorption performance, with the resulting composites exhibiting high-efficiency MB removal. Adsorption followed the Langmuir isotherm and pseudo-second-order kinetics, indicating monolayer chemisorption, while mechanistic analysis revealed synergistic contributions from electrostatic attraction, π-π stacking, and physical entrapment. This work establishes clear structure–property relationships linking carbon dimensionality to hydrogel network topology and adsorbent function, providing a rational design strategy for advanced adsorbents and a theoretical foundation for efficient dye wastewater remediation.

## 4. Materials and Methods

### 4.1. Materials

Acrylamide (AM, CP, ≥98%), sodium alginate [SA, CP, viscosity ≥ 20 mPa·s (10 g/L, 20 °C), pH 6–8 (10 g/L, 25 °C)], ammonium persulfate (APS, AR, ≥98%), graphene oxide (GO, 99%), carboxylated carbon nanotubes (CNT, 95%), hydrochloric acid (36.0~38.0%), ferric sulfate (AR, ≥99.5%), sodium hydroxide (AR, ≥96%), sodium chloride (AR, ≥99.5%), potassium chloride (AR, ≥99.5%), ammonium chloride (AR, ≥99.5%), calcium chloride (AR, ≥96.0%), magnesium chloride hexahydrate (AR, ≥98%) and methylene blue (MB, 98%) were purchased from Sinopharm Chemical Reagent Co., Ltd., Shanghai, China. All chemicals were used without further purification. Rice husk biochar (BC) was purchased from Henan Lize Environmental Protection Technology Co., Ltd., Zhengzhou, China. Deionized water, tap water, lake water (Huicui Lake, Dongying, China), and river water (Guangli River, Dongying, China) were used for the experiments.

### 4.2. Preparation of the Dual Physically Crosslinked Hydrogel

**Figure 18:**
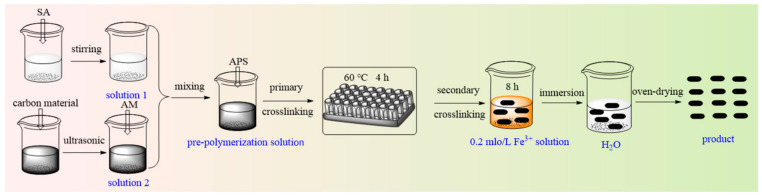
Synthesis process of dual physically crosslinked hydrogel.

In this experiment, the amounts of all components except the carbon material were determined based on previous laboratory studies. The preparation process is illustrated in Figure 18. Specifically, 0.6 g of SA was dissolved in 10 mL of deionized water to form Solution 1. Then, 0.2 g of GO was dispersed in 15 mL of deionized water, followed by the addition of 5.0 g of AM into the GO dispersion. The mixture was sonicated until AM was completely dissolved, yielding Solution 2. Solutions 1 and 2 were combined, after which 1 mL of APS solution (0.05 g/mL) was added via a microsyringe. The mixture was stirred for 10 min and then degassed under vacuum to remove bubbles. The resulting solution was poured into a mold, sealed with plastic wrap, and reacted at 60 °C for 4 h to form the primarily crosslinked hydrogel. This hydrogel was subsequently immersed in a 0.2 mol/L Fe^3+^ solution for 4 h to achieve secondary crosslinking. The dual physically crosslinked hydrogel was then washed in deionized water for 8 h, with the water replaced each hour. The sample was cut into small pieces and dried at 80 °C for 3 h, yielding the final dry product of the dual physically crosslinked hydrogel. A series of carbon material composite dual physically crosslinked hydrogels was prepared by varying the type (GO, CNT, or BC) and amount (0.2 g, 0.4 g, 0.6 g, 0.8 g, or 1.0 g) of the carbon material.

### 4.3. Mechanical Property Determination

Mechanical characterization was performed on an electronic universal testing machine (HDV, HANDPI, Leqing, China). Tensile properties were measured using rectangular specimens (30 × 10 × 5 mm^3^ at a speed of 80 mm/min), while compression tests employed cylindrical samples (20 mm height × 10 mm diameter) at 8 mm/min. All tests were carried out in triplicate, with results expressed as mean values.

### 4.4. Swelling Performance

The dry samples were immersed in deionized water or different pH solutions for 8 h. After removing and blotting off surface moisture, the swelling degree (S) was calculated using Equation (10) [40]:

(10) $$S\;=\frac{M_{2}-M_{1}}{M_{1}}$$

where M_1_ and M_2_ denote the mass of the hydrogel before and after swelling, respectively.

### 4.5. Determination of pH_pzc_

A measurement of 0.1 g of hydrogel was placed in a series of 0.01 mol/L NaCl aqueous solutions with different initial pH (pH_0_) values. After standing at room temperature for 48 h, the final pH (pH_f_) of each solution was measured using a pH meter. The change in pH (ΔpH = pH_f_ − pH_0_) was calculated. The point of zero charge (pH_pzc_) of the hydrogel was preliminarily estimated by plotting the relationship between pH_0_ and ΔpH.

### 4.6. Adsorption Experiments

**Figure 19:**
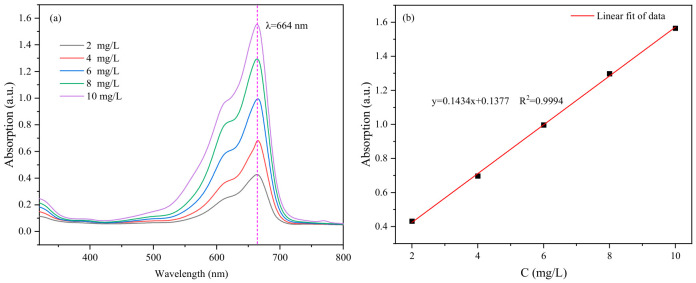
(**a**) UV-Vis absorption spectrum of MB and (**b**) calibration curve for MB concentration.

A 1000 mg/L MB stock solution was prepared. A series of standard solutions with concentrations ranging from 2 to 10 mg/L was obtained by diluting the stock solution. The absorbance of these solutions was measured at the maximum absorption wavelength of MB (664 nm) using a 722S UV-Vis spectrophotometer. A calibration curve was established based on the absorbance data, as shown in Figure 19. For the adsorption experiments, the post-adsorption dye solutions were diluted appropriately to ensure their concentrations fell within the linear range of the calibration curve, allowing the determination of dye concentrations via the standard curve equation.

The adsorption capacity (Q, mg/g) and removal efficiency (R, %) were determined using Equations (11) and (12), respectively [41].

(11) $$Q=\frac{(C_{0}{-C}_{e})V}{m}$$

(12) $$R(\%)=\frac{\left(C_{0}-C_{e}\right)}{C_{0}}$$

where C_0_ and C_e_ represent the initial and equilibrium concentrations (mg/L) of MB, m denotes the mass (g) of the dry hydrogel, and V corresponds to the solution volume (L).

#### 4.6.1. Effect of Carbon Material Loading

Hydrogels with varying carbon material loadings (0.2–1.0 g of GO, CNT, or BC) were evaluated for MB adsorption. In standard tests, 10.0 mg of dry hydrogel was introduced into 50 mL of MB solution (100 mg/L, unadjusted pH) and shaken at 25 °C until equilibrium. The equilibrium adsorption capacity was then determined.

#### 4.6.2. pH Effect

The pH-dependent adsorption was examined across a range of 3.0–11.0. Samples (10.0 mg) were immersed in 50 mL of MB solution (100 mg/L) with pH adjusted using 0.1 M NaOH/HCl. After reaching equilibrium at 25 °C, the residual concentration was measured to quantify pH influence.

#### 4.6.3. Temperature Effect

To investigate the effect of temperature, 10.0 mg of dry hydrogel was introduced into 50 mL of MB solution (100 mg/L, unadjusted pH) and shaken at 25, 35, 45, and 55 °C, respectively, until equilibrium.

#### 4.6.4. Adsorption Isotherms

Isotherm experiments employed MB solutions with concentrations ranging from 50 to 250 mg/L. Each trial used 10.0 mg hydrogel in 50 mL solution (natural pH: 6.5) at 25 °C. Equilibrium data were analyzed to construct adsorption isotherms and determine maximum uptake.

#### 4.6.5. Adsorption Kinetics

Kinetic profiles were obtained by monitoring MB removal at 10 min intervals. Dry hydrogel (10.0 mg) was exposed to 50 mL of 100 mg/L MB solution at 25 °C. Temporal concentration changes were tracked until equilibrium to establish kinetic parameters.

#### 4.6.6. Performance in Natural Waters

Practical efficacy was tested using tap, lake, and river water spiked with MB (100 mg/L). For each water matrix, 10.0 mg hydrogel was added to 50 mL solution and agitated at 25 °C. Post-equilibrium analysis determined the removal efficiency in different aqueous environments.

#### 4.6.7. Recyclability

The MB-saturated hydrogel samples were regenerated by immersion in anhydrous ethanol under continuous shaking at 25 °C for 8 h. After desorption, the hydrogels were thoroughly rinsed with deionized water to remove residual solvent and released dye molecules. Subsequently, the samples were equilibrated in a 0.2 mol/L Fe^3+^ aqueous solution for 1 h to reconstruct the ionic crosslinked network. Finally, the regenerated hydrogels were washed with deionized water, dried at 80 °C for 3 h, and then subjected to subsequent adsorption–desorption cycle tests.

### 4.7. Structural Characterization

FTIR spectroscopy was performed on a NICOLET iN10 spectrometer (Thermo Fisher Scientific, Waltham, MA, USA) over the wavenumber range of 4000–400 cm^−1^ using the KBr pellet method. Surface morphology was examined using a FEI QUANTA FEG 450 scanning electron microscope (FEI, New York, NY, USA) at an accelerating voltage of 10 kV. Prior to observation, all samples were sputter-coated with gold to improve conductivity. Crystal structure was analyzed by X-ray diffraction (XRD) on a Rigaku D/Max-II-2500VB2/PC diffractometer (Rigaku, Tokyo, Japan) using Cu Kα radiation at 40 kV and 100 mA. Data were collected over a 2θ range of 5–80° with a scanning speed of 5°/min. Chemical states were characterized via X-ray photoelectron spectroscopy (XPS) on a Thermo Scientific ESCALAB 250Xi system using monochromatic Al Kα radiation. All binding energies were calibrated using the C 1s peak (284.8 eV) as reference. Data analysis was performed using version 6.9 of avantage software. Three-dimensional surface topography was assessed using an OLS5000-SFA confocal laser scanning microscope (CLSM, Olympus, Tokyo, Japan) equipped with a 408 nm laser and operated at a magnification of 100×. The Brunauer–Emmett–Teller (BET) data of the samples were tested by a nitrogen adsorption instrument, AutoSorb iQ2 (Quantachrome, Boynton Beach, FL, USA).

## Figures and Tables

**Table 1:** Fitting results of adsorption isotherm.

Hydrogels	Langmuir				Freundlich		
	Q_m_ (mg·g^−1^)	K_b_ (L·mg^−1^)	R_L_	R^2^	K_f_ [g^(−1)^·mg^(1−1/n)^·L^(−1/n)^]	1/n	R^2^
BC-H	476.19	0.0533	0.0698~0.2729	0.9851	129.7	0.2353	0.9727
GO-H	485.44	0.0399	0.0911~0.3339	0.9794	96.39	0.2937	0.9728
CNT-H	487.80	0.0548	0.0680~0.2674	0.9937	113.8	0.2728	0.9871

**Table 2:** Fitting results of knetic models.

Hydrogels	Pseudo-First Order Model			Pseudo-Second Order Model			Intra-Particle Diffusion Model		
	Q_e,cal_ (mg·g^−1^)	k_1_ (min^−1^)	R^2^	Q_e,cal_ (mg·g^−1^)	k_2_ × 10^4^ (g·mg^−1^·min^−1^)	R^2^	K_1_	K_2_	K_3_
BC-H	250.06	0.0485	0.9903	323.62	2.53	0.9971	43.49	15.64	3.91
GO-H	265.28	0.0593	0.9709	310.56	2.63	0.9902	42.39	6.06	1.94
CNT-H	366.61	0.0751	0.9645	337.84	2.79	0.9968	46.43	13.54	2.28

**Table 3:** Thermodynamic parameters.

Adsorbents	ΔG(kJ/mol)				ΔH (kJ/mol)	ΔS (J/mol·K)
	298 K	308 K	318 K	328 K		
BC-H	−4.83	−4.65	−4.48	−4.30	−10.11	−17.72
GO-H	−4.68	−4.56	−4.43	−4.31	−8.36	−12.35
CNT-H	−5.12	−4.86	−4.60	−4.34	−12.91	−26.13

**Table 4:** BET and BJH parameters of GO-H hydrogel before and after MB adsorption.

	BET Surface Area (m^2^·g^−1^)	BET Adsorption Average Pore Width (nm)	BJH Adsorption Average Pore Diameter (nm)	Single Point Adsorption Total Pore Volume (cm^3^/g)	BJH Adsorption Cumulative Volume (cm^3^/g)
GO-H	1.1538	15.01	18.52	0.003459	0.003258
GO-H-MB	0.9216	10.39	17.51	0.002997	0.002693
GO-H (after four adsorption–desorption cycles)	0.5057	7.97	14.2	0.001008	0.000593

**Table 5:** Comparison of the adsorption capacity for MB with different adsorbents.

Adsorbent	Conditions	Q_max_ (mg·g^−1^)	Reference
BC-g-PHEAAm/Fe_3_O_4_	600 mg/L	200	[32]
Alg/Biochar10 hydrogel	12 mg/L; pH 7; 25 °C	214.6	[33]
PAM/BC	20 mg/L; pH 11	23.53	[27]
BC-H	100 mg/L; 25 °C; pH 10	411.5	This study
PGS–CS–GO	100 mg/L; pH 7	179	[34]
PVA/PCMC/GO/bentonite	250 mg/L; pH 10; 30 °C	172.14	[35]
CS/PHPA/GO	100 mg/L; pH 7; 30 °C	468	[36]
GO-H	100 mg/L; 25 °C; pH 11	410.6	This study
CNT hybrid hydrogel	0.1 mg/L; 20 °C	33.45	[37]
CS-g-GEL/SWCNTs	20 mg/L; 25 °C; pH 9	69.92	[38]
TG/CFCNT HBNC	50 mg/L; 40 °C; pH 11	647	[39]
CNT-H	100 mg/L; 25 °C; pH 11	422.8	This study

## Data Availability

All data generated or analyzed during this study are included in the published article. The data presented in this study are available on request from the corresponding author.
